# Purification of a single-photon nonlinearity

**DOI:** 10.1038/ncomms12578

**Published:** 2016-08-30

**Authors:** H. Snijders, J. A. Frey, J. Norman, M. P. Bakker, E. C. Langman, A. Gossard, J. E. Bowers, M. P. van Exter, D. Bouwmeester, W. Löffler

**Affiliations:** 1 Huygens-Kamerlingh Onnes Laboratory, Leiden University, P.O. Box 9504, 2300 RA Leiden, The Netherlands; 2 Department of Physics, University of California, Santa Barbara, California 93106, USA; 3 Department of Electrical and Computer Engineering, University of California, Santa Barbara, California 93106, USA

## Abstract

Single photon nonlinearities based on a semiconductor quantum dot in an optical microcavity are a promising candidate for integrated optical quantum information processing nodes. In practice, however, the finite quantum dot lifetime and cavity-quantum dot coupling lead to reduced fidelity. Here we show that, with a nearly polarization degenerate microcavity in the weak coupling regime, polarization pre- and postselection can be used to restore high fidelity. The two orthogonally polarized transmission amplitudes interfere at the output polarizer; for special polarization angles, which depend only on the device cooperativity, this enables cancellation of light that did not interact with the quantum dot. With this, we can transform incident coherent light into a stream of strongly correlated photons with a second-order correlation value up to 40, larger than previous experimental results, even in the strong-coupling regime. This purification technique might also be useful to improve the fidelity of quantum dot based logic gates.

## Abstract (web summary)

Single-photon optical nonlinearity is possible using an optical cavity to create strong coupling between a cavity mode and a two-level quantum system. Here, the authors demonstrate it is also possible in the weak-coupling regime by using quantum interference in a polarization-degenerate cavity.

## Graphical abstract

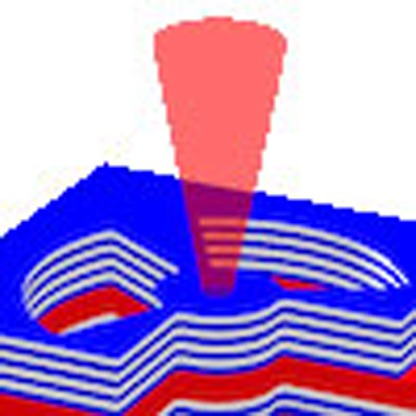

Single-photon nonlinearities enabled by quantum two-level systems are essential for future quantum information technologies, as they are the building block of quantum photonics logic gates^1^, deterministic entanglers of independent photons^2^ and for coupling distant nodes to form a quantum network^3^. Near-unity fidelity interaction of photons with a two-level system such as an atom or quantum dot (QD) is enabled by embedding it into an optical cavity^4^. Next, the electronic and photonic states become bound and form the dressed states^5^ of cavity quantum electrodynamics (CQED). A hallmark of single-photon nonlinearities is the modification of the photon statistics of a quasi-resonant weak coherent input beam^6^: the transmitted photon statistics can become antibunched due to the photon blockade effect^1^,^7^,^8^. The anharmonicity of the Jaynes–Cummings (JC) ladder^9^,^10^,^11^ can also be used to reach the regime of photon tunnelling^6^,^12^ where the single-photon component is reduced, leading to enhanced photon correlations, or the appearance of *N*>1 multiphoton ‘bundles'^13^,^14^.

In terms of the second-order photon correlation function *g*^2^(0), values up to ∼2 (refs 15, 16, 17, 18) have been obtained experimentally with QDs, which hardly exceeds even the classical case of thermal light following Bose statistics of *g*^2^(0)=2. In atomic systems with much longer coherence times, values up to ∼50 have been obtained^6^ and it is known^19^ that strict two-photon light sources exhibit diverging *g*^2^(0) if the two-photon flux is reduced. Most related QD experiments to date have been operating in the strong-coupling regime of CQED, which is considered to be essential due to its photon-number-dependent energy structure^6^,^17^,^18^. In the weak-coupling regime, the energy structure is not resolved and it is an open question whether photon-number-dependent JC effects can still be observed^20^. The strong coupling regime, however, requires a small optical mode volume, which in turn makes it extremely hard to achieve polarization degeneracy of the fundamental cavity mode. This is due to unavoidable deviations from the ideal shape and intrinsic birefringence^21^,^22^ on the GaAs platform, precluding implementation of deterministic polarization-based quantum gates^2^,^23^,^24^.

Here we show, using a nearly polarization-degenerate cavity in the weak coupling CQED regime, that we can transform incident coherent light into a stream of strongly correlated photons with *g*^2^(0)=25.7±0.9, corresponding to ≳40 in the absence of detector jitter. The polarization-degenerate cavity enables us to choose the incident polarization *θ*_in_=45° such that both fine-structure split QD transitions along 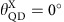 and 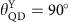 are excited, and we can use a postselection polarizer behind the cavity (*θ*_out_) to induce quantum interference of the two transmitted orthogonal polarization components (Fig. 1a). This leads to the appearance of two special postselection polarizer angles 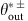 (depending on sample parameters), which can be used to restore perfect QD contrast (red curves in Fig. 1b). This compensates fully for reduced QD cavity coupling due to finite QD lifetime and QD cavity coupling strength, leading to complete suppression of transmission of the single-photon component in the low excitation limit. The transmission of higher-photon number states remains largely intact, allowing us to observe in Fig. 1c the strongest photon correlations to date in a solid-state system, reaching the range of strongly coupled atomic systems^6^. In the following, a detailed experimental and theoretical investigation of this effect, which can be seen as a purification of a single-photon nonlinearity, will be presented.

## Results

### Device structure

Our device consists of self-assembled InAs/GaAs QDs embedded in a micropillar Fabry–Perot cavity grown by molecular beam epitaxy^25^ (see Supplementary Fig. 1 and Supplementary Note 1). The QD layer is embedded in a P–I–N junction, separated by a 35 nm-thick tunnel barrier from the electron reservoir, to enable tuning of the QD resonance frequency by the quantum-confined Stark effect. For transverse mode confinement and to achieve polarization degenerate cavity modes, we first ion-etch micropillars of large diameter (35 μm) and slightly elliptical shape, then we use wet-chemical oxidation of an AlAs layer^26^ to prepare an intra-cavity lens for transverse-mode confinement^27^, avoiding loss by surface scattering at the side walls. Finally, we fine-tune the cavity modes by laser induced surface defects^28^,^29^ to obtain a polarization mode splitting much smaller than the cavity linewidth.

### Device parameters and theoretical model

The system we study here is tuned to contain a single neutral QD within the cavity linewidth. The excitonic fine-structure splitting leads to 4.8 GHz splitting between the orthogonally polarized QD transitions at 0° 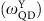 and 90° 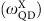. The fundamental cavity modes show a residual polarization splitting of 4 GHz (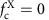 GHz, 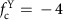 GHz) and the cavity axes are rotated by 5° with respect to the QD axes. To determine further system parameters, we model our QD cavity system by a two-polarization JC Hamiltonian coupled to the incident coherent field and take care of cavity and QD dissipation by the quantum master equation formalism^30^,^31^. We compare experiment and theory for six different input–output polarizer settings to faithfully determine the model parameters, these measurements were performed for an input power of 100 pW to avoid saturation effects^32^. We obtain (see Supplementary Fig. 2 and Supplementary Note 2) a cavity decay rate *κ*=105±3 ns^−1^, QD relaxation rate *γ*_||_=1.0±0.4 ns^−1^, QD pure dephasing γ*=0.6±0.0 ns^−1^ and QD cavity coupling rate *g*=14±0.1 ns^−1^; from which we can calculate the device cooperativity 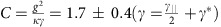. As 4*g*^2^/(*γ*^2^+*κ*^2^)=0.07, our system operates in the weak-coupling bad-cavity regime of cavity QED.

### Resonant photon correlation spectroscopy

**Figure 1:**
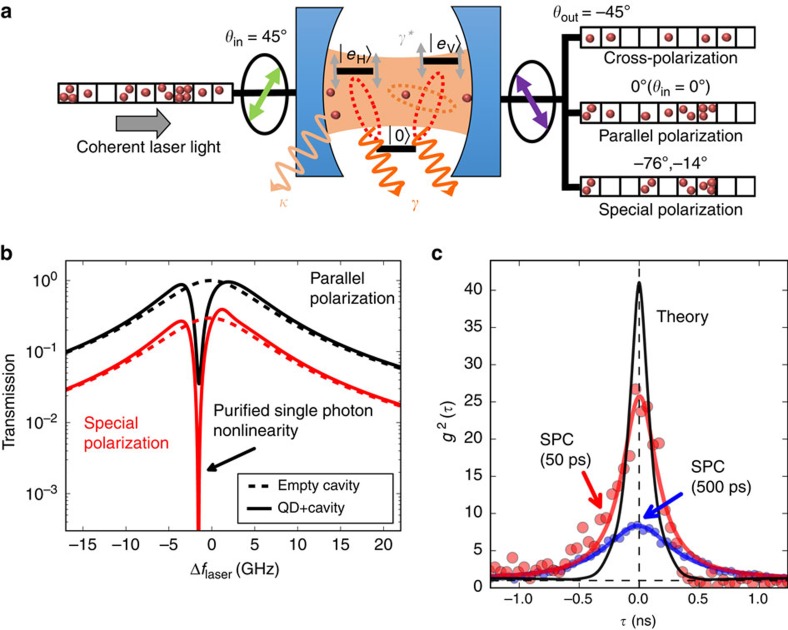
The purification technique. (**a**) Cartoon of the experiment: polarization pre- and postselection in a resonant transmission CQED experiment enables tuning of the photon statistics from antibunched to bunched. (**b**) Theoretical resonant transmission spectra for coherent light with mean photon number <<1, with and without the QD, comparing the conventional case (parallel polarizers) with the case of special polarization postselection along 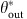: close to one of the QD resonances, single-photon transmission is perfectly suppressed, despite the finite lifetime and cavity coupling of the QD transition. (**c**) Second-order correlation function for the special polarization angle case, comparing theory and experiment using two different sets of single photon counters (SPCs) with different timing jitter, 50 ps and 500 ps.

We use a narrowband (100 kHz) laser to probe the system and study the transmitted light (Fig. 1a), as a function of laser frequency and postselection polarizer angle behind the cavity. For each set of parameters, we measure the resonantly transmitted light intensity and its second-order photon correlation function *g*^2^(*τ*) using a Hanbury Brown Twiss setup. The discrete nature of the QD levels leads to a strongly nonlinear response of the system depending on the incident photon number distribution; we operate at low intensities to avoid saturation effects. We show here only data for an incident polarization *θ*_in_=45°, under which angle both QD transitions are equally excited.

**Figure 2:**
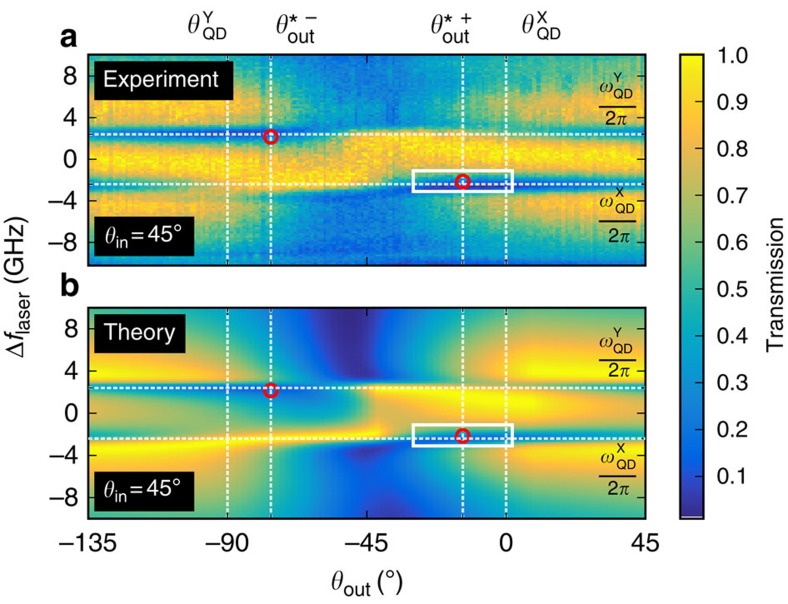
Coherent probing of the QD cavity system. Experimental (**a**) and theoretical (**b**) false colour plot of the columnwise normalized optical transmission as a function of the laser detuning Δ*f*_Laser_ and the polarization *θ*_out_ (*θ*_in_=45°). The fine-split QD transition frequencies are at 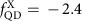 GHz and 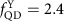 GHz. The red circles indicate the special polarization conditions; the white square indicates the area explored in Fig. 3.

First, we compare experimental and theoretical resonant transmission measurements in Fig. 2, where the coherent light transmittivity as a function of the laser detuning and orientation of the output polarizer angle *θ*_out_ is shown. For clarity, we have normalized the traces for each polarization setting. The horizontal lines indicate the QD fine structure split transitions 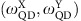, the black circles indicate regions of low transmission and the vertical dashed lines the special polarization angles 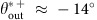, 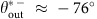. From comparision of both panels in Fig. 2, we find excellent agreement between experiment and theory.

**Figure 3:**
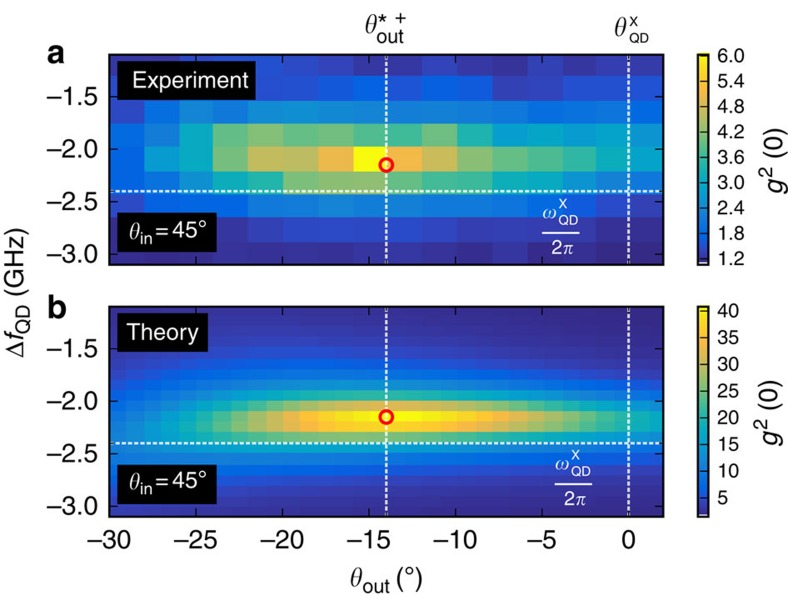
Photon bunching and purification. Experimental (**a**) and theoretical (**b**) data of the second-order correlation function as a function of the QD frequency and output polarization (*θ*_out_), taken in the area marked with a white rectangle in Fig. 2. The vertical dashed lines indicate the special polarization angle and the QD axis, and the horizontal line indicate the QD resonance frequency.

Now we perform photon correlation measurements; instead of tuning the laser, we now tune the QD, the reference are the cavity modes. As the cavity linewidth is large compared with the QD tuning range in Fig. 3, there is nearly no difference compared with tuning the laser. Experimentally, using an external electric field to tune the QD via the quantum confined Stark effect is much more robust than laser frequency tuning. Figure 3 shows the false-colour map of *g*^2^(0) as function of output polarization *θ*_out_ and QD detuning. We see clearly that the enhanced bunching occurs under the special polarization condition in the low-transmittivity regions indicated in Fig. 2. This is expected as in weak coherent light beams, the *P*_1_ single-photon component is dominating and removal thereof should lead to enhanced bunching. The theoretical simulation (Fig. 3b) shows a maximal photon bunching of *g*^2^(0)≈40. Compared with this, the experimentally observed photon correlations are less (*g*^2^(0)≈6), which is due to the detector response: Fig. 3a was recorded with a 500 ps timing-jitter detector, if we repeat the measurement at the special polarization angle with a 50 ps timing-jitter detector (the corresponding *g*^2^(*τ*) measurements are compared in Fig. 1c), we obtain *g*^2^(0)=25.7±0.9. Both results agree very well to the convolution of the theoretically expected *g*^2^(*τ*) with the detector responses (Fig. 1c; see also Supplementary Fig. 3 and Supplementary Note 3).

## Discussion

We have shown by experiment and theory that the reduced fidelity of a QD nonlinearity, caused by imperfect QD-cavity coupling, can be strongly enhanced by pre- and post-selection of specific polarization states. This enables transformation of a weak coherent input beam into highly bunched light with *g*^2^(0)≳40, a value that has not been reached before, not even in the strong coupling regime. How is it possible to reach such high photon correlations, how does the polarization-based purification technique work?

**Figure 4:**
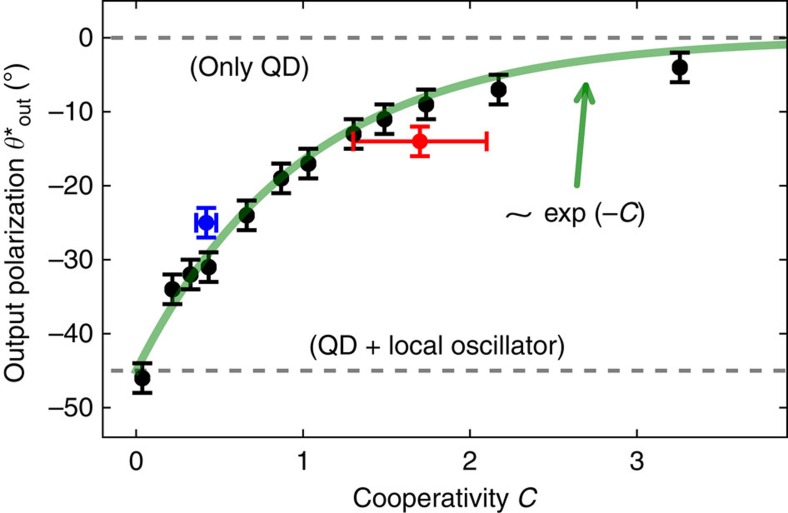
Special polarization and cooperativity. Black: numerically determined special polarization angle, where photon bunching in transmission is maximized, as a function of the cooperativity *C*, which in turn is modified by varying only the QD lifetime *γ*_||_. The green curve is given by the phenomenological expression −45°exp (−*C*): in the limit of high *C*, the QD alone can efficiently filter out single-photon states, leading to photon bunching. However, for low cooperativity *C*, it is advantageous to mix the quantum-dot scattered light with a ‘local oscillator' provided by orthogonal polarization. The error bars (all s.d.) are due to numerical errors in optimizating the laser frequency. The red data point corresponds to the sample presented here; the blue data corresponds to another device with lower cooperativity.

We consider incident light with a frequency in the vicinity of one of the QD resonances, say 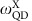, and let us decompose the electromagnetic field transmitted through the cavity in two orthogonally polarized components: the signal Field *E*_S_ polarized along the QD resonance polarization 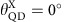 and the local oscillator *E*_LO_, which has interacted with an empty cavity, because it is polarized orthogonally to 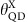. Now, we consider three cases: (i) efficient interaction of the QD with incident light (cooperativity *C*>1), (ii) intermediate interaction (*C*≈1) and (iii) weak interaction (*C*→0). The special polarization angles for various cooperativities are shown in Fig. 4.

In case (i), the QD leads to a nearly complete removal of the single-photon component from the incident coherent light polarized along the QD polarization: these photons are in principle perfectly reflected from the cavity and we simply have to detect along the same axis (
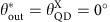
, see Fig. 4) to observe strong photon correlations. A significant proportion of higher photon number states are transmitted. As the second-order correlation function can be expressed in terms of the photon number distribution as 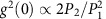 (ignoring *N*>2 photon number states), which for *P*_2_<<*P*_1_ and *P*_*N*>2_<<*P*_2_, this leads to diverging photon correlations such as 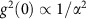 if the single-photon component is attenuated as *P*_1_→*αP*_1_.

Now in case (ii), for realistic systems, the finite lifetime of the QD transition and/or limited QD-cavity coupling *g* leads to a reduced cooperativity: even in the low-excitation limit, not every single-photon state is filtered out. Therefore, the signal field *E*_S_ contains a fraction of coherent light reducing the photon bunching along the QD polarization 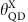, compare Fig. 3. This effect has been called ‘self-homodyning' in literature^33^,^34^. With the purification technique, we now rotate the postselection polarizer to interfere a portion of the local oscillator field *E*_LO_ with the signal field, leading to the superimposed field 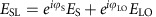
35. The polarizer angle controls the relative intensity of the two components and we can control the transmission phases *ϕ*_s_ and *ϕ*_LO_ by adjusting the laser frequency, because the phases vary strongly in the vicinity of the QD and cavity resonances. We simply have to choose the local oscillator intensity that it matches the intensity of the portion of *E*_S_ and adjust the phases for destructive interference. The result is that we detect in transmission mainly the single-photon filtered portion of *E*_S_, which leads to very high photon correlations in the transmitted light despite limited cooperativity.

Finally, in case (iii) for *C*→0, only a vanishing fraction of the photons have interacted with the QD. We have to tune the postselection polarizer to −45° to destructively interfere nearly equal amounts of *E*_S_ and *E*_LO_ to observe enhanced photon correlations. This case is similar to that recently investigated in ref. 36, where (weak) photon bunching is observed for a relative phase of *π* (*ϕ*_s_−*ϕ*_LO_=*π*). We have a high-finesse (*F*≈800) cavity and significant cooperativity, which enables us to observe much stronger photon correlations (Supplementary Fig. 4 and Supplementary Note 4).

The special postselection angle 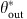 and laser frequency have to be optimized numerically in principle, because pure dephasing cannot be taken care of in a semiclassical model. Despite this, we found that the special polarization angle shows approximately a very simple dependency on the cooperativity: 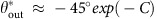, see Fig. 4, which agrees well to our intuitive explanation here.

As a last point, we analyse the strong photon bunching in terms of the photon number distribution *P*_n_. We use our theoretical model to determine *P*_n_, as direct experimental determination thereof is strongly complicated by its sensitivity to loss. However, also the simulation of narrow-band photon number Fock input states is challenging in the quantum master model^37^. Therefore, we use coherent input light and analyse the intra-cavity light in terms of its polarized photon number distribution, taking care of quantum interference at the postselection polarizer acting on the intra-cavity field. This is an approximation, because imperfect transmission through the cavity reshapes *P*_n_. We found that the photon statistics *P*_n_ can be calculated best by projection on the required Fock states using polarization-rotated Fock space ladder operators 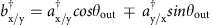 and tracing out the undesired polarization component afterwards. With the numerically^31^ calculated steady-state density matrix operator *ρ* of our system (Supplementary Note 2), we obtain the photon number distribution after the polarizer:


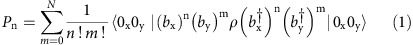


**Figure 5:**
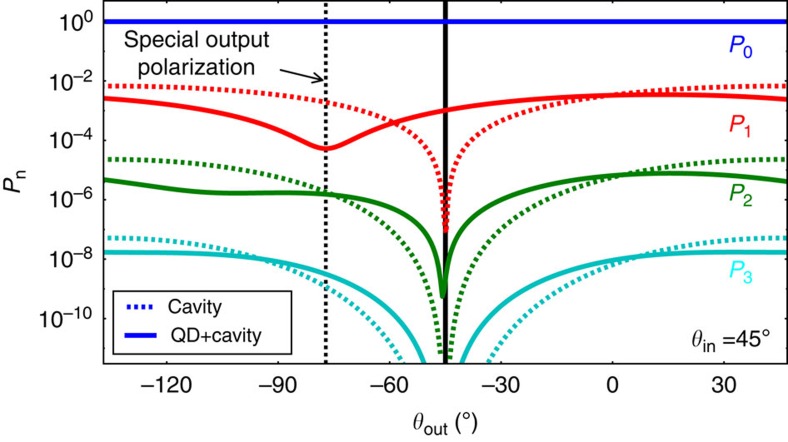
Photon number distributions. Calculated photon number distribution after the polarizer, with (through curves) and without (dashed curves) coupling to the QD in the cavity, the laser frequency is set to one of the QD resonances. With QD, we clearly see the photon-number-dependent shift of the transmission dip. Only the photon number distribution of the detected polarization component is shown; therefore, the total number of photons in case with QD can exceed the case without QD due to polarization conversion by the dot. For clarity, pure dephasing has been neglected here, making the special polarization angle different from the other simulations and experimental results.

Figure 5 shows the four lowest photon number probabilities as a function of the polarizer angle *θ*_out_, for the case with and without QD. In the empty-cavity case we see, as expected, lowest transmission under the cross-polarization condition (*θ*_out_=−45°). For the case with the QD, we observe a photon-number-dependent shift of the transmission dip. At the special polarization angle 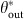, we see that the one-photon component reaches a minimum, while the higher-photon number states do not, which explains the enhanced photon bunching enabled by the purification technique.

It is important to note that also the two-photon transmission dip (*P*_2_) is not exactly at cross-polarization, which suggests the following intuitive explanation: apparently, in the photon number basis, the different Fock states pick up a different phase during transmission through the QD cavity system. In the weak-coupling regime, but often also in the strong coupling regime, the individual JC dressed states cannot be resolved spectrally, because g≲*k*. However, the CQED system is still photon-number sensitive, which implies lifetime-dependent JC effects in the weak coupling regime: the decay rate of the CQED system increases with the number of photons in the cavity^20^,^38^. As consequence, higher photon-number states have a modified interaction cross-section and experience a reduced phase shift. The dip in *P*_2_ in Fig. 5 is already very close to the cross-polarization angle *θ*_out_=−45°, whereas the dips for higher photon number states *P*_n>2_ are indistinguishable from *θ*_out_=−45°.

In conclusion, we found that the nonlinear response of a lossy cavity QD system can be strongly enhanced by postselection of a particular polarization state. This leads to interference between Fock states that experienced different modifications by the QD nonlinearity and results in strong photon correlations of the transmitted light. As the underlying effect, interference of the two polarizations modes leads to high-fidelity cancellation of the single-photon transmission for the special polarization postselection condition. By correlating the results with a theoretical model, we found indications of photon-number sensitive JC physics in the weak coupling regime of CQED.

### Data availability

All relevant data are available on request.

## Additional information

**How to cite this article:** Snijders, H. *et al*. Purification of a single-photon nonlinearity. *Nat. Commun.* 7:12578 doi: 10.1038/ncomms12578 (2016).

## Supplementary Material

Supplementary Information Supplementary Figures 1-4, Supplementary Notes 1-4 and Supplementary References
