# Supplementary material for: Purification of a single-photon nonlinearity
Source: Nat Commun. 2016 Aug 30;7:12578. doi: 10.1038/ncomms12578 (PMC5013554; doi:10.1038/ncomms12578)
Supplement: Supplementary Information — Supplementary Figures 1-4, Supplementary Notes 1-4 and Supplementary References [file ncomms12578-s1.pdf]

SUPPLEMENTARY FIGURE 1

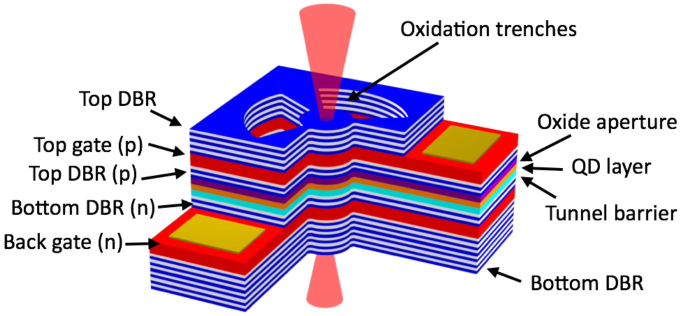

Supplementary Figure 1: Scheme of the device. The red cones indicate light transmitted through the cavity.

SUPPLEMENTARY FIGURE 2

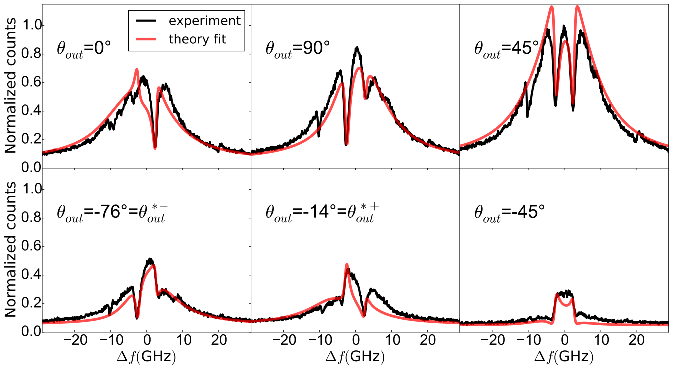

Supplementary Figure 2: Parameter estimation. Experimental data (black) and the theoretical fit (red). The input polarization was set to $\theta_{in} = 45°$ and θ^{*+}_{out} and θ^{*-}_{out} indicate the special polarization angles.

SUPPLEMENTARY FIGURE 3

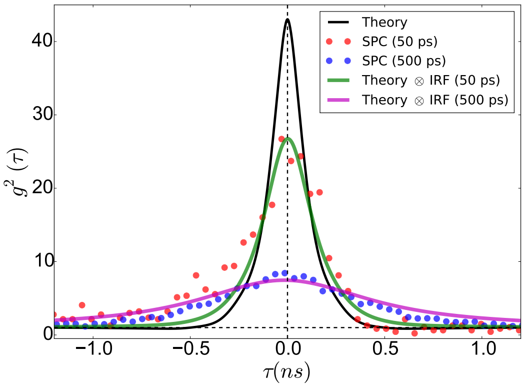

Supplementary Figure 3: Detector response. Comparison of the theoretical data with and without taking care of detector jitter, and the experimental $g^2(\tau)$ data for our QD. The agreement between theory and experiment is excellent.

SUPPLEMENTARY FIGURE 4

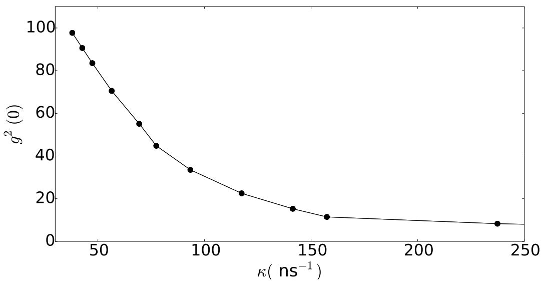

Supplementary Figure 4: influence of the cavity quality. Shown is the calculated maximal (i.e., for special polarizer angles) $g^2(0)$ for different cavity decay rates. A good cavity with low κ is needed in order to reach the extreme bunching values $g^2(0)$.

SUPPLEMENTARY NOTE 1 DEVICE STRUCTURE

The samples under study (Supplementary Fig. 1) are grown by molecular beam epitaxy on a GaAs [100] substrate. Two distributed Bragg reflectors (DBR) surround a $\sim 5\lambda$ thick cavity containing in the center InGaAs self-assembled quantum dots (QDs) and an oxide aperture for transverse confinement. The top DBR mirror consists of 26 pairs of $\lambda/4$ thick GaAs / $Al_{0.90}Ga_{0.10}As$ layers, while the bottom mirror has 13 pairs of GaAs / AlAs layers and 16 pairs of GaAs / $Al_{0.90}Ga_{0.10}As$ layers. The aperture is made of a 10 nm thick AlAs layer which is embedded between 95 nm $Al_{0.83}Ga_{0.17}As$ and 66 nm thick $Al_{0.75}Ga_{0.25}As$. After wet chemical oxidation this enables an intra-cavity lens for transverse mode confinement. The quantum dots are separated by an undoped 35 nm thick GaAs tunnel barrier from the n-doped GaAs:Si (2.0×10^{18} cm^{-3}) electron reservoir.

SUPPLEMENTARY NOTE 2 THEORETICAL MODEL

A. Jaynes - Cummings quantum master equation

We describe the QD-cavity system via an extended version of a two level system in an optical cavity, which is driven by a classical coherent laser field. Albeit our cavities have only a small polarization splitting of the fundamental modes, we take full care of it. The quantum description, based on the application of a unitary transformation to transform the Hamiltonian from a time dependent to a time independent form and the rotating wave approximation, results in the following Hamiltonian ($\hbar = 1$)[1, 2]:

$$\begin{aligned} H \; = \; & \left(\omega_L - \omega_c^X\right) \hat{a}_X^\dagger \hat{a}_X + \left(\omega_L - \omega_c^Y\right) \hat{a}_Y^\dagger \hat{a}_Y + \left(\omega_L - \omega_{QD}^X\right) \hat{\sigma}_X^\dagger \hat{\sigma}_X + \left(\omega_L - \omega_{QD}^Y\right) \hat{\sigma}_Y^\dagger \hat{\sigma}_Y \\ & + g_Y \left(\hat{\sigma}_Y \hat{b}_Y^\dagger + \hat{\sigma}_Y^\dagger \hat{b}_Y\right) + g_X \left(\hat{\sigma}_X \hat{b}_X^\dagger + \hat{\sigma}_X^\dagger \hat{b}_X\right) \\ & + \frac{\eta}{2} \left[e_x' \left(\hat{a}_X^\dagger + \hat{a}_X\right) + e_y' \left(\hat{a}_Y^\dagger + \hat{a}_Y\right)\right] + \frac{1}{2}(\omega_c^X - \omega_c^Y) \left[\hat{a}_X^\dagger \hat{a}_Y + \hat{a}_Y^\dagger \hat{a}_X\right] \end{aligned} \tag{1}$$

Here $\omega_c^{X/Y}$ are the cavity resonance frequencies of the polarized cavity modes, and $\omega_{QD}^{X/Y}$ are the fine-structure-split QD transition frequencies. $\hat{a}_{X/Y}^\dagger$ is the photon creation operator for a photon in X/Y polarization, and $\hat{\sigma}_{X/Y}^\dagger$ creates an X/Y polarized neutral exciton. The terms with coupling constants $g_{X/Y}$ describe the interaction between a QD transition and the cavity field, which is rotated into the QD polarization basis by $\hat{b}_X = \hat{a}_X cos\,\phi + \hat{a}_Y sin\,\phi$ and $\hat{b}_Y = -\hat{a}_X sin\,\phi + \hat{a}_Y cos\,\phi$, where ϕ is the rotation angle. This Hamiltonian is designed for a cavity with a small polarization splitting. The last term describes the driving of the cavity by an external linearly polarized coherent laser field, where η^2 is proportional to the incident intensity [3], and the Jones vector $\left(e_x', e_y'\right)$ describes the incident light polarization.

Next we write down a quantum master equation for our Hamiltonian and include Lindblad-type dissipation for the

cavity decay rate κ, the population relaxation rate $\gamma_{||}$ and the total pure dephasing rate γ^*.

$$\frac{d\rho}{dt} = \mathfrak{L}\rho = -i\left[\hat{H}, \rho\right] + \sum_{j=X,Y} \frac{\kappa}{2}\mathfrak{D}[\hat{a}_j]\rho + \frac{\gamma_{||}}{2}\mathfrak{D}[\hat{\sigma}_j]\rho + \frac{\gamma^*}{4}\mathfrak{D}[\hat{\sigma}_{zj}]\rho, \tag{2}$$

Where ρ is the density matrix of the QD-cavity system, $\mathfrak{L}$ is the Liouvillian superopererator for QD-cavity density matrix and $\mathfrak{D}[\hat{o}]\rho \equiv 2\hat{o}\rho\hat{o}^\dagger - \hat{o}^\dagger\hat{o}\rho - \rho\hat{o}^\dagger\hat{o}$ results in Lindblad type dissipation. Here $\hat{\sigma}_{zj}$ is defined as $\frac{1}{2}\left(\hat{\sigma}_j^\dagger\hat{\sigma}_j - \hat{\sigma}_j\hat{\sigma}_j^\dagger\right)$. This Lindblad-type master equatition in Eq. 2 is based on the validity of several additional approximations (see for instance [4]), where we point out a few: (1) full separability of the system and the environment at $t = 0$, and (2) the state of the environment does not change significantly under interaction with the system, i.e., the interaction is weak, and the system and environment remain separable throughout the evolution. Last, we assume (3) that the environment has no memory on the time scale of the system (Markov approximation). Those approximations are justified as we only discuss photonic interaction with the environment here, which is very weak. We are interested in the steady state solution for ρ, and solve $\mathfrak{L}\rho = 0$, using the numerical methods provided by the software package QUTIP [5].

B. Transmission and photon correlations

The cavity transmittivity is calculated by $T = \text{Tr}\left[\rho_0\left(e_1\hat{a}_X^\dagger + e_2\hat{a}_Y^\dagger\right)(e_1\hat{a}_X + e_2\hat{a}_Y)\right] = \text{Tr}\left(\rho_0\hat{a}^\dagger\hat{a}\right)$, where (e_1, e_2) describes the output polarizer Jones vector, and ρ_0 is the steady-state density matrix of the system. We investigate the photon correlations by calculating the second-order correlation function, which is independent of mirror loss and can therefore be calculated directly from the intracavity photon operators $\langle\hat{a}^\dagger\hat{a}\rangle$. The second-order correlation function is given by $g^2(\tau) = \frac{\langle\hat{a}^\dagger(0)\hat{a}^\dagger(\tau)\hat{a}(\tau)\hat{a}(0)\rangle}{\langle\hat{a}^\dagger(0)\hat{a}(0)\rangle^2}$ with the time dependent photon creation operator $\hat{a}^\dagger(\tau)$. In order to solve the time dependence of the operator $\hat{a}^\dagger(\tau)$, we assume that the effect of the operator $\mathfrak{L}$ is small [6] and the eigenvalues are nondegenerate, which allows us to write $\hat{a}^\dagger(\tau)$ as $\hat{a}^\dagger e^{\mathfrak{L}\tau}$.

C. Model parameters

For estimation of the parameters, we fit the theory above discussed to the experimental transmission data for 6 different output polarizations for $\theta_{in} = 45°$, i.e., both QD transitions are excited. The result (Supplementary Fig. 2) shows good agreement between experiment (blue curve) and theory (red curve). We obtain the best-fit parameters $\kappa = 105 \pm 3$ ns^{-1}, $g = 14 \pm 0.1$ ns^{-1}, $\gamma^{||} = 1.0 \pm 0.4$ ns^{-1}, $\gamma^* = 0.6 \pm 0.01$ ns^{-1}, $f_{QD}^{X/Y} = -2.4/2.4$ GHz. The residual cavity polarization splitting is 4 GHz ($f_c^X = 0$ GHz, $f_c^Y = -4$ GHz, where the $\{X, Y\}$ axes are rotated by $\phi = 5°$ with respect to the quantum dot axes. We note that another quantum dot is visible within the cavity resonance, compare Supplementary Fig. 2 for $\theta_{out} = 90°$ at around -10 GHz; but since it is much less strongly coupled to the cavity mode it can be neglected.

SUPPLEMENTARY NOTE 3 DETECTOR RESPONSE

In order to show that the true two-photon correlations are much stronger than the raw experimental data suggests, we now present details on the convolution of the theoretical $g^2(\tau)$ data with the single photon counter (SPC) detector response. We use two detectors with 50 ps and 500 ps detector jitter, which was determined by measuring photon correlations of a picosecond Ti:Sapphire laser oscillator. As shown in Supplementary Fig. 3 we observe very good agreement between the convoluted theoretical prediction and the experimental data for our QD. Since count rates were high, we could also perform the experiment with a less sensitive 50 ps jitter detector, which again agrees very well to theory. This clearly shows that our $g^2(\tau)$ measurements are severly reduced by the detector jitter of the single photon counters, but that we can fully deconvolute this effect.

SUPPLEMENTARY NOTE 4 PHOTON CORRELATIONS AND CAVITY QUALITY

Here we show that the cavity is essential to obtain such strong photon correlations as we have observed experimentally. For this we conduct numerical simulations for various cavity decay rates κ. In order to isolate the effect of κ, we have to optimize for each value of κ the laser frequency and the output polarization to find the special polarization angle and thereby the maximum in the $g^2(0)$ landscape. Next to this we also need to keep the internal mean photon number constant by increasing the incident laser power for a higher value of κ. In order to do this we optimized the power coupling parameter η for each value of κ, so that the mean photon number of the outgoing light (for parallel polarization $\theta_{in} = \theta_{out} = 45°$) on the cavity resonance for an empty cavity remains constant. The result is shown in Supplementary Fig. 4: In the case of almost no cavity (large κ), only very small $g^2(0)$ values are obtainable, while in good cavities (small κ), extreme values of $g^2(0)$ are possible. The other parameters for simulation of Supplementary Fig. 4 are similar to those of the device in the main text.

SUPPLEMENTARY REFERENCES

[1] E.T. Jaynes and F.W. Cummings, "Comparison of quantum and semiclassical radiation theories with application to the beam maser," Proc. IEEE **51**, 89 (1963).

[2] Christophe Arnold, Justin Demory, Vivien Loo, Aristide Lemaître, Isabelle Sagnes, Mikhaïl Glazov, Olivier Krebs, Paul Voisin, Pascale Senellart, and Loïc Lanco, "Macroscopic rotation of photon polarization induced by a single spin," Nat. Commun. **6**, 6236 (2015).

[3] Jing Tang, Weidong Geng, and Xiulai Xu, "Quantum Interference Induced Photon Blockade in a Coupled Single Quantum Dot-Cavity System," Sci. Rep. **5**, 9252 (2015).

[4] J.R. Johansson, P.D. Nation, and Franco Nori, "QuTiP: An open-source Python framework for the dynamics of open quantum systems," Comput. Phys. Commun. **183**, 1760–1772 (2012).

[5] J. R. Johansson, P. D. Nation, and Franco Nori, "QuTiP 2: A Python framework for the dynamics of open quantum systems," Comput. Phys. Commun. **184**, 1234–1240 (2013).

[6] C.W. Gardiner and Peter Zoller, *Quantum Noise* (Springer, 2004).
